# Protonated deca­fluoro­benzo­phenone and the deca­fluoro­benzo­phenone–arsenic penta­fluoride adduct

**DOI:** 10.1107/S2053229625007697

**Published:** 2025-09-23

**Authors:** Erik Uran, Matic Lozinšek

**Affiliations:** a Extreme Conditions Chemistry Laboratory (ECCL K2), Jožef Stefan Institute, Jamova cesta 39, 1000 Ljubljana, Slovenia; b Jožef Stefan International Postgraduate School, Jamova cesta 39, 1000 Ljubljana, Slovenia; University of Oxford, United Kingdom

**Keywords:** superacid medium, protonation, deca­fluoro­benzo­phenone, AsF_5_ adduct, crystal structure, protonated ketone

## Abstract

The reaction of deca­fluoro­benzo­phenone [perfluoro­benzo­phenone, (C_6_F_5_)_2_CO] with AsF_5_ in anhydrous HF yields the protonated salt [bis­(2,3,4,5,6-penta­fluoro­phen­yl)methyl­idene]oxidanium hexa­fluorido­arsenate, (C_6_F_5_)_2_COH^+^[AsF_6_]^−^, whereas its reaction with AsF_5_ in SO_2_ affords the Lewis acid–base adduct deca­fluoro­benzo­phenone–arsenic penta­fluoride, (C_6_F_5_)_2_CO·AsF_5_. In both com­pounds, the deca­fluoro­benzo­phenone moiety exhibits an elongated C=O bond [1.274 (2) and 1.2526 (15) Å in the salt and adduct, respectively]. The crystal structure of (C_6_F_5_)_2_COH^+^[AsF_6_]^−^ features a short O—H⋯F hy­dro­gen bond between the cation and the anion, and the crystal structure of (C_6_F_5_)_2_CO·AsF_5_ represents a rare example of a ketone coordinated to the strong Lewis acid AsF_5_.

## Abstract 2

Protonated deca­fluoro­benzo­phenone (perfluoro­benzo­phenone) and its corresponding AsF_5_ adduct were synthesized by reaction of the ketone with AsF_5_ using anhydrous HF and liquid SO_2_, respectively, as solvents, and their crystal structures were determined.

## Introduction

Protonation of small mol­ecules plays an important role in investigating and elucidating the reaction mechanisms of acid-catalysed organic reactions. These reactive cations are typically generated in superacidic media (Olah *et al.*, 2009 ▸) and a wide range of com­pounds has been shown to undergo pro­ton­ation under such conditions. Examples include nitriles (Haiges *et al.*, 2016 ▸; Goetz *et al.*, 2016 ▸), organic azides (Saal *et al.*, 2020 ▸), carb­oxy­lic acids (Saal *et al.*, 2023 ▸; Hollenwäger *et al.*, 2024 ▸), acyl halides (Steiner *et al.*, 2024*a* ▸), amides (Axhausen *et al.*, 2013 ▸; Saal *et al.*, 2023 ▸), esters (Hollenwäger *et al.*, 2025 ▸) and amino sulfonic acids (Bockmair *et al.*, 2024 ▸).

The protonation of aldehydes and ketones in solution has been studied in detail by low-tem­per­a­ture NMR spectroscopy. For example, the protonation of aliphatic aldehydes (Olah *et al.*, 1967*b* ▸) and aliphatic ketones (Olah *et al.*, 1967*a* ▸) was studied in the superacidic media HSO_3_F–SbF_5_–SO_2_, proton­ated alicyclic ketones in HSO_3_F–SbF_5_–SO_2_ and HSO_3_F–SO_2_ (Olah & Calin, 1968 ▸), protonated aceto­phenones in HSO_3_F (Birchall & Gillespie, 1965 ▸) and benzo­phenones in HSO_3_F–SbF_5_ (Sekuur & Kranenburg, 1966 ▸; van der Linde *et al.*, 1968 ▸). The reaction of hexa­fluoro­acetone in an HF–SbF_5_ mixture, however, results in HF addition to the ketone, followed by protonation, yielding a rare example of a protonated alcohol (Minkwitz & Reinemann, 1999 ▸).

Several crystallographically characterized examples of pro­ton­ated ketones (Childs *et al.*, 1982 ▸, 1990 ▸; Chadda *et al.*, 1986 ▸; Stuart *et al.*, 2017 ▸; Schickinger *et al.*, 2018 ▸) and proton­ated aldehydes (Hwang *et al.*, 2010 ▸; Heo *et al.*, 2011 ▸; Hayatifar *et al.*, 2014 ▸; Stuart *et al.*, 2017 ▸) have been reported. The crystal structure of protonated benzo­phenone has been reported in the form of [HCB_11_H_5_Cl_6_]^−^ carborane (Stasko *et al.*, 2002 ▸) and [TaF_6_]^−^ salts (Marchetti *et al.*, 2007 ▸), both featuring a hy­dro­gen-bonded dimer, [(C_6_H_5_)_2_C=O—H⋯O=C(C_6_H_5_)_2_]^+^.

The protonation of deca­fluoro­benzo­phenone, (C_6_F_5_)_2_CO, in solution was previously studied in H_2_SO_4_ and HSO_3_F (Cham­bers & Spring, 1969 ▸), and in HF–SbF_5_–SO_2_ClF mixtures (Olah & Mo, 1973 ▸) by NMR spectroscopy. Research on Lewis acid–base adducts of AsF_5_ with ketones remains scarce (Stuart *et al.*, 2019 ▸) and no crystal structures containing a C_2_CO–AsF_5_ fragment have been reported in the Cambridge Structural Database (CSD, Version 6.00, April 2025; Groom *et al.*, 2016 ▸).

In this work, the synthesis and structural characterization of the protonated deca­fluoro­benzo­phenone (perfluoro­benzo­phe­none) salt (C_6_F_5_)_2_C=OH^+^[AsF_6_]^−^ and a Lewis acid–base adduct of arsenic penta­fluoride and deca­fluoro­benzo­phenone, *i.e.* (C_6_F_5_)_2_CO·AsF_5_, are reported.

## Experimental

### Synthesis and crystallization

**Figure 1:**
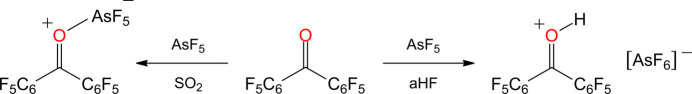
Synthesis of the (C_6_F_5_)_2_COH^+^[AsF_6_]^−^ salt and the (C_6_F_5_)_2_CO·AsF_5_ adduct.

**Figure 2:**
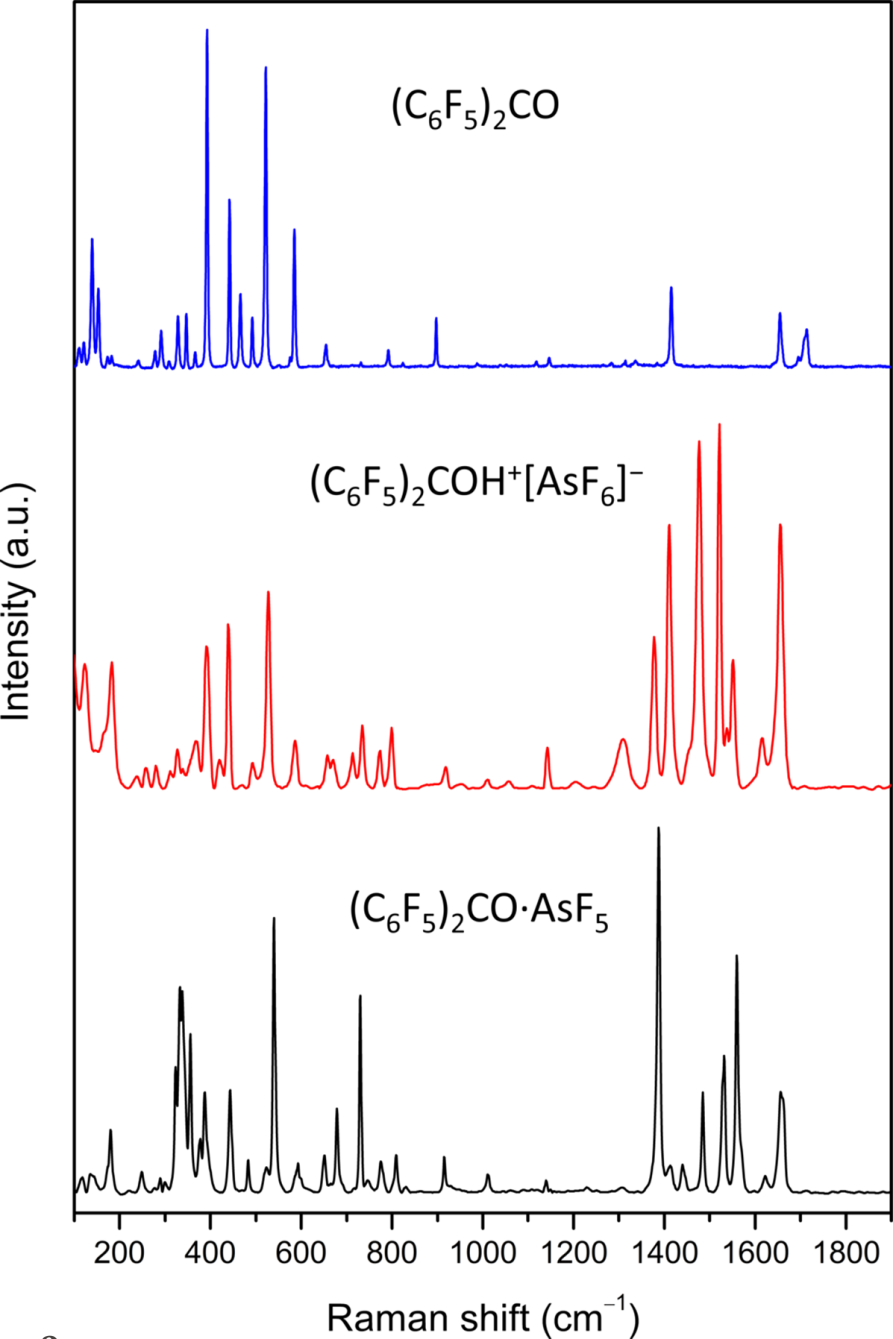
The low-tem­per­a­ture Raman spectra of (C_6_F_5_)_2_CO (top), (C_6_F_5_)_2_COH^+^[AsF_6_]^−^ (middle) and (C_6_F_5_)_2_CO·AsF_5_ (bottom) measured at −90, −90 and −93 °C, respectively.

**Figure 3:**
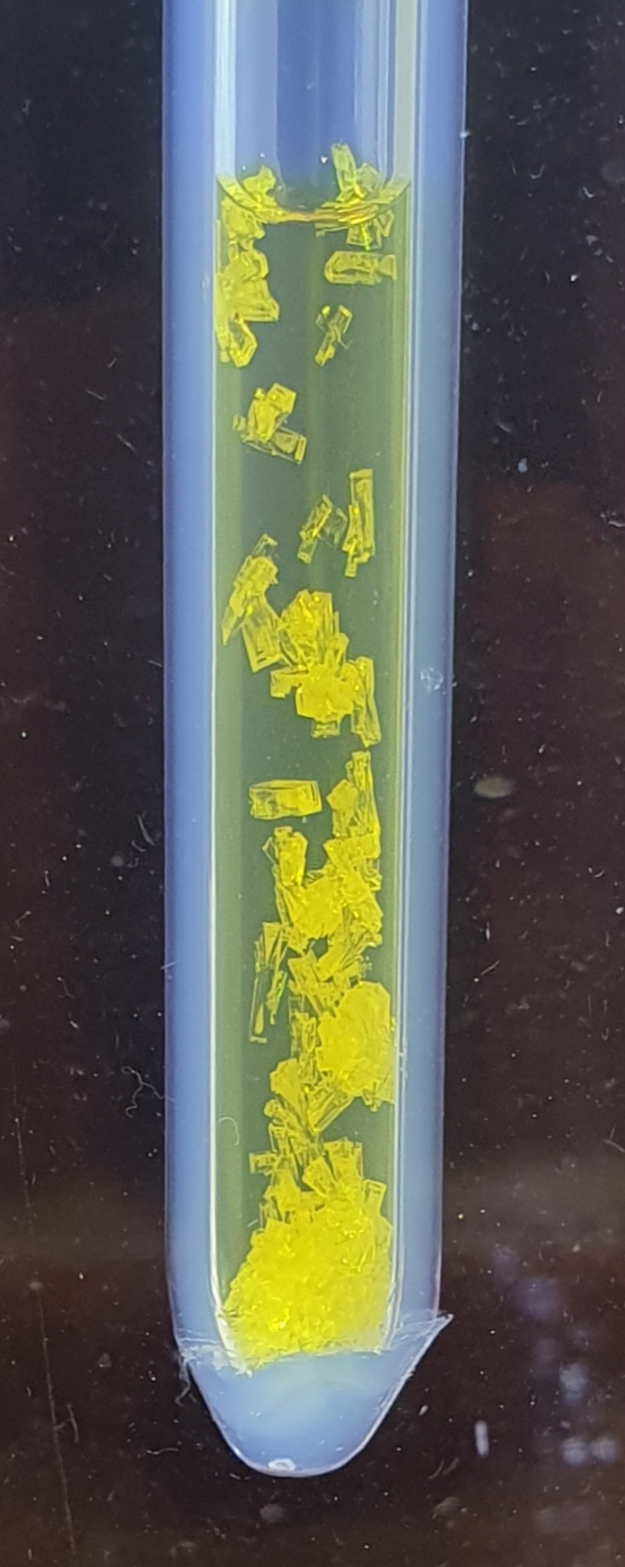
Canary-yellow crystals of (C_6_F_5_)_2_COH^+^[AsF_6_]^−^ that formed upon cooling a solution of (C_6_F_5_)_2_CO and AsF_5_ in anhydrous HF to about −10 °C using an ethanol bath. The outer diameter of the FEP tube is 6 mm.

A fluorinated ethyl­ene propyl­ene (FEP) vessel (outer di­am­eter: 6 mm; inner diameter: 4.6 mm), equipped with an aluminium-encased polychloro­tri­fluoro­ethyl­ene (PCTFE) valve, was passivated with fluorine overnight prior to use. In a nitro­gen-filled glovebox (Vigor SG1200/750E–SG1500/750E; H_2_O < 0.02 ppm, O_2_ < 2 ppm), the vessel was loaded with (C_6_F_5_)_2_CO (23 mg, 0.064 mmol; Thermo Scientific, 97%). Anhydrous HF (0.2 ml; Linde, 99.995%; stored over K_2_NiF_6_) was then condensed into it using a vacuum line designed for handling elemental fluorine, resulting in a suspension of (C_6_F_5_)_2_CO at room tem­per­a­ture. Upon addition of AsF_5_ (0.137 mmol), synthesized as described previously (Mazej & Žemva, 2005 ▸), a yellow precipitate formed that dissolved com­pletely upon warming to room tem­per­a­ture. Slow cooling to −30 °C yielded canary-yellow crystals of (C_6_F_5_)_2_COH^+^[AsF_6_]^−^. The solvent aHF was removed under dynamic vacuum at −78 °C, leaving a yellow crystalline solid. Another synthesis was performed on a slightly larger scale [113 mg, 0.312 mmol (C_6_F_5_)_2_CO; 0.4 ml aHF; 0.936 mmol AsF_5_] (Figs. 1–3 ▸ ▸ ▸).

For the synthesis of (C_6_F_5_)_2_CO·AsF_5_, (C_6_F_5_)_2_CO (14 mg, 0.039 mmol) was dissolved in dry SO_2_ (0.3 ml; Ruše, stored over CaH_2_ in a glass bulb) at room tem­per­a­ture. AsF_5_ (0.168 mmol) was added at −196 °C. Upon thawing of the SO_2_–AsF_5_ mixture, a yellow coloration appeared immediately. The vessel was then stored at −78 °C under a nitro­gen overpressure (∼1000 Torr). Large yellow crystals of (C_6_F_5_)_2_CO·AsF_5_ formed over a period of ten days. Volatiles, SO_2_ and excess AsF_5_ were removed under dynamic vacuum between −78 and −50 °C, yielding a yellow solid (Figs. 1 ▸ and 2 ▸).

A crystalline sample was quickly deposited from the FEP reaction vessel into an aluminium trough of the low-tem­per­a­ture crystal-mounting apparatus, designed and employed for mounting highly reactive moisture-sensitive noble-gas com­pounds (Lozinšek *et al.*, 2021 ▸; Motaln *et al.*, 2024 ▸), which was cooled using a stream of cold nitro­gen to tem­per­a­tures ranging from −80 to −100 °C. Suitable crystals were selected under a stereomicroscope and mounted on the tip of a MiTeGen loop using Fomblin oil (Z25, SynQuest) (Motaln *et al.*, 2025 ▸). The loop assembly was picked up with cryo-pin tongs cooled to −196 °C and quickly transferred to the magnetic holder on the goniometer head, where the crystal was protected by a cold nitro­gen stream at 100 K.

### Refinement

Crystal data, data collection and structure refinement details are summarized in Table 1 ▸. The position of the H atom in the crystal structure of (C_6_F_5_)_2_COH^+^[AsF_6_]^−^ was located in a difference electron-density map and refined freely, including its isotropic displacement parameter (Cooper *et al.*, 2010 ▸).

### Raman spectroscopy

Raman spectroscopy was performed using either a Horiba Jobin Yvon LabRAM HR spectrometer coupled with an Olympus BXFM-ILHS microscope or a Bruker Senterra II confocal Raman microscope equipped with a Linkam LTS420 low-tem­per­a­ture stage. Red (633 and 785 nm) and green (532 nm) lasers were tested, with the laser power adjusted to the sample. The Raman spectra of (C_6_F_5_)_2_COH^+^[AsF_6_]^−^ and (C_6_F_5_)_2_CO·AsF_5_ exhibited fluorescence regardless of the excitation wavelength used. The low-tem­per­a­ture (−90 °C) spectrum of solid (C_6_F_5_)_2_CO (Fig. 2 ▸), which is in good agreement with the previously published Raman spectrum (Anandhi & Umapathy, 2000 ▸), was recorded using the Bruker spectrometer with a 785 nm red laser at 1.5 cm^−1^ resolution. Low-tem­per­a­ture Raman spectra of the (C_6_F_5_)_2_COH^+^[AsF_6_]^−^ salt and the (C_6_F_5_)_2_CO·AsF_5_ adduct (Fig. 2 ▸) were acquired on the Horiba system using a 633 nm red laser at 4 cm^−1^ resolution from crystalline material protected by a cold nitro­gen stream of the crystal-mounting apparatus. Background subtraction from the spectra was performed using the Bruker *OPUS8.7* software suite, with 15 iterations of concave rubber-band correction and 50 baseline points.

## Results and discussion

To facilitate a com­parison of structural changes in the deca­fluoro­benzo­phenone moiety upon protonation and coordination to AsF_5_, the crystal structure of (C_6_F_5_)_2_CO was re­de­termined at 100 K (Table 1 ▸; Figs. S1 and S2 in the supporting information). The current structural parameters [C=O = 1.197 (4) Å; C—CO = 1.498 (3) Å; C—C = 1.373 (3)–1.392 (3) Å; C—F = 1.331 (3)–1.341 (3) Å] are in excellent agreement with the previously reported crystal structure measured at 153 K [C=O = 1.201 (8) Å; C—CO = 1.489 (6) Å; C—C = 1.361 (6)–1.403 (5) Å; C—F = 1.329 (5)–1.351 (4) Å; Schwarzer *et al.*, 2004 ▸]. Deca­fluoro­benzo­phenone crystallizes in the Sohncke space group *C*2 and the mol­ecule exhibits axial chirality owing to sterically hindered rotation of the C_6_F_5_ rings about the C_ar­yl_—C(O)—C_ar­yl_ bonds (Farmer & Walker, 1967 ▸). Similarly, chiral crystallization has also been reported for achiral benzo­phenone (Matsumoto *et al.*, 2016 ▸).

The synthetic procedure for the preparation of (C_6_F_5_)_2_COH^+^[AsF_6_]^−^ and (C_6_F_5_)_2_CO·AsF_5_ involved the reaction of (C_6_F_5_)_2_CO with AsF_5_ in anhydrous HF (aHF) and in liquid SO_2_ sol­vent, respectively (Fig. 1 ▸). Although deca­fluoro­benzo­phenone was poorly soluble in aHF, the addition of AsF_5_ resulted in its dissolution at room tem­per­a­ture, accom­panied by the development of a yellow coloration. Upon cooling the reaction mixture, canary-yellow plank-shaped crystals began to form (Fig. 3 ▸). Slow removal of the aHF solvent yielded a crystalline canary-yellow solid, identified as (C_6_F_5_)_2_COH^+^[AsF_6_]^−^.

**Figure 4:**
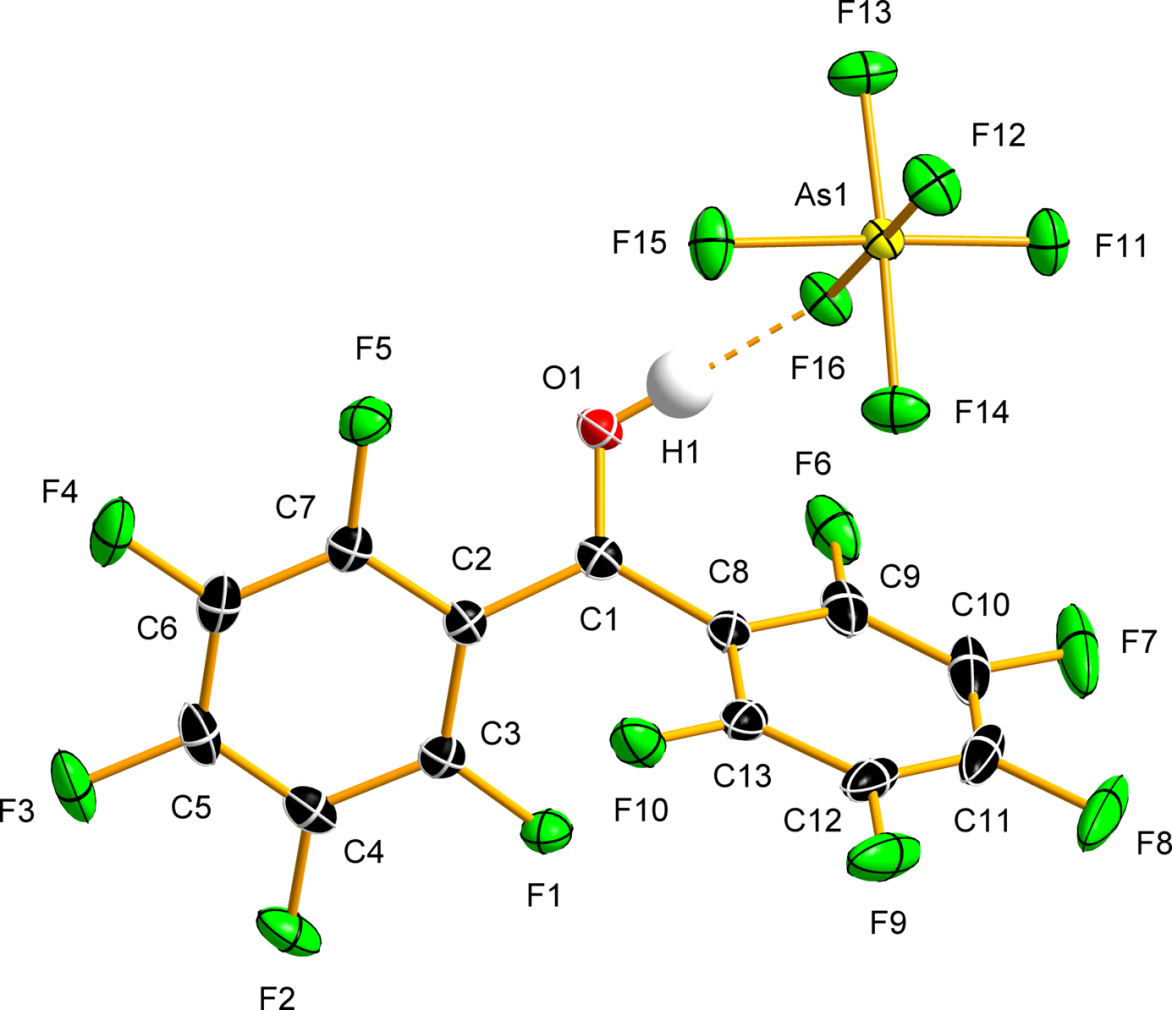
The asymmetric unit with the atom-labelling scheme in the crystal structure of (C_6_F_5_)_2_COH^+^[AsF_6_]^−^. The O—H⋯F hy­dro­gen bond is shown as an orange dashed line. Displacement ellipsoids are de­picted at the 50% probability level.

The protonated salt (C_6_F_5_)_2_COH^+^[AsF_6_]^−^ crystallizes in the monoclinic space group *P*2_1_/*n* and contains one (C_6_F_5_)_2_COH^+^ cation and one [AsF_6_]^−^ anion in the asymmetric unit (Table 1 ▸ and Figs. 4 ▸ and S3).

In (C_6_F_5_)_2_COH^+^[AsF_6_]^−^ (Fig. 4 ▸), the C=O bond length [1.274 (2) Å] is elongated com­pared to that in (C_6_F_5_)_2_CO [1.197 (4) Å], and is com­parable to values observed in the protonated ketones: acetone, C_3_H_6_OH^+^ [1.271 (3) and 1.273 (3) Å]; adamantan-2-one, C_10_H_14_OH^+^ [1.274 (2) Å]; and cyclo­penta­none, C_5_H_8_OH^+^ [1.266 (3) and 1.267 (2) Å] (Stuart *et al.*, 2017 ▸). Similar C=O bond lengths have been reported for the crystal structures of the hemiprotonated benzo­phenone salts [(C_6_H_5_)_2_CO]_2_H^+^[CHB_11_H_5_Cl_6_]^−^ [1.274 (4) Å; Stasko *et al.*, 2002 ▸] and [(C_6_H_5_)_2_CO]_2_H^+^[TaF_6_]^−^ [1.259 (3) Å; Marchetti *et al.*, 2007 ▸]. The C—C(=O) bonds in (C_6_F_5_)_2_COH^+^ [1.443 (2) and 1.455 (2) Å] are shorter than the corresponding bonds in (C_6_F_5_)_2_CO [1.498 (3) Å]. The remaining C—F and C—C bonds in the (C_6_F_5_)_2_COH^+^ cation [C—C = 1.370 (2)–1.406 (2) Å; C—F = 1.322 (2)–1.334 (2) Å] remain within the range of those observed in the crystal structure of (C_6_F_5_)_2_CO. The angle between the plane normals of the two arene rings [76.58 (6)°] is very similar to that in (C_6_F_5_)_2_CO [77.22 (9)°]. The angles between the arene-ring-plane normals and the plane normal of the carbonyl-bond-containing C_2_C=O fragment [36.93 (7) and 44.90 (7)°] are smaller than the corresponding value observed in the crystal structure of (C_6_F_5_)_2_CO [47.97 (7)°].

The crystal structure exhibits a short hy­dro­gen bond between the (C_6_F_5_)_2_COH^+^ cation and the [AsF_6_]^−^ anion (Table 2 ▸). The H atom lies only slightly out of the C_2_C=O plane [0.14 (3) Å]. The O⋯F hy­dro­gen-bond distance [2.5279 (16) Å] is shorter than those observed in the crystal structures of [PnF_6_]^−^ (Pn = As or Sb) salts of protonated ketones – acetone, cyclo­penta­none and adamantan-2-one [2.560 (3)–2.6233 (16) Å; Stuart *et al.*, 2017 ▸] – due to the greater Brønsted acidity of the perfluorinated conjugate acid (C_6_F_5_)_2_COH^+^. The hy­dro­gen-bonded As—F bond is elongated [As—F(H) = 1.7853 (10) Å; As—F = 1.6943 (10)–1.7237 (11) Å], resulting in a distortion of the [AsF_6_]^−^ anion from idealized octa­hedral geometry. Inter­estingly, similar As—F_bridging_ bond lengths have been observed in metal com­plexes where the [AsF_6_]^−^ anion is coordinated to a metal centre, for example, in [Mg(KrF_2_)_4_(AsF_6_)_2_] [1.7856 (14) and 1.7965 (13) Å] (Lozinšek *et al.*, 2017 ▸).

The supporting information includes an additional crystal structure determination of (C_6_F_5_)_2_COH^+^[AsF_6_]^−^ employing Ag *K*α radiation, providing results essentially similar to those presented in the text.

**Figure 5:**
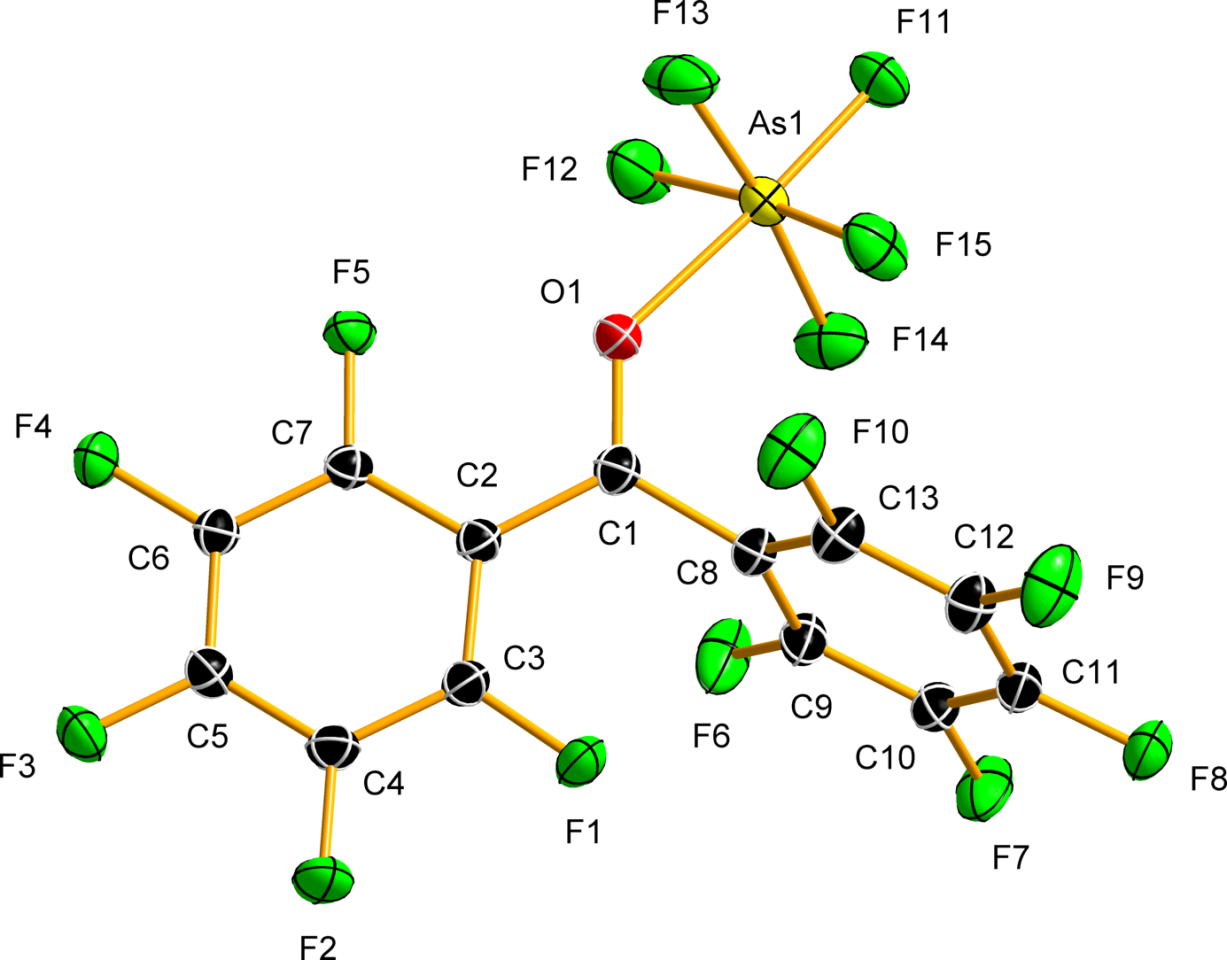
The asymmetric unit with the atom-labelling scheme in the crystal structure of adduct (C_6_F_5_)_2_CO·AsF_5_. Displacement ellipsoids are de­picted at the 50% probability level.

The reaction of deca­fluoro­benzo­phenone with an excess of AsF_5_ in SO_2_ at low tem­per­a­ture (Fig. 1 ▸) resulted in the formation of a yellow solution, from which large yellow plate-like crystals of the Lewis acid–base adduct (C_6_F_5_)_2_CO·AsF_5_ crystallized at −78 °C over a period of ten days. The com­pound crystallizes in the ortho­rhom­bic space group *Pbca* (Table 1 ▸) and represents a rare crystallographically characterized example of a ketone coordinated to AsF_5_ (Figs. 5 ▸ and S4).

The C=O bond length [1.2526 (15) Å] in the crystal structure of the adduct is elongated com­pared to that in (C_6_F_5_)_2_CO [1.197 (4) Å], although to a lesser extent than in the protonated salt [1.274 (2) Å]. A similar C=O bond length was observed in the benzo­phenone adduct with TaCl_5_ [1.265 (3) Å; Marchetti *et al.*, 2007 ▸], in the adducts of BF_3_ with ethyl­ene carbonate [1.2486 (14) Å], dimethyl carbonate [1.2559 (14) Å], diethyl carbonate [1.2562 (18) Å] and γ-butyrolactone [1.2541 (16) Å] (Bodin *et al.*, 2023 ▸), and in the amide moiety of capsaicin [1.2459 (19) Å; Lozinšek, 2025 ▸]. The elongation of the C=O bond is accom­panied by a significant shortening of the *anti*-C—C(=O) bond [1.4549 (16) Å], whereas the *syn*-C—C(=O) bond length remains virtually unchanged [1.4879 (16) Å] com­pared to the corresponding distance observed in (C_6_F_5_)_2_CO [1.498 (3) Å]. All other bond lengths in the (C_6_F_5_)_2_CO moiety within (C_6_F_5_)_2_CO·AsF_5_ [C—C = 1.3788 (17)–1.4133 (15) Å; C—F = 1.3162 (13)–1.3359 (15) Å] are similar to those observed in (C_6_F_5_)_2_COH^+^[AsF_6_]^−^ and (C_6_F_5_)_2_CO. The angle between the plane normals of the two arene rings [81.57 (4)°] differs from those in the crystal structures of (C_6_F_5_)_2_CO and (C_6_F_5_)_2_COH^+^[AsF_6_]^−^. Of the two rings, one is almost coplanar with the plane formed by the C_2_C=O moiety [9.44 (5)°], while the other is nearly perpendicular [75.63 (5)°].

The O—As bond [1.9897 (10) Å] in the crystal structure of (C_6_F_5_)_2_CO·AsF_5_ is substanti­ally longer than that reported for the adduct of Roesky’s ketone, S_2_N_2_CO·AsF_5_ [1.879 (7) Å; Gieren *et al.*, 1980 ▸], and shorter than the O—As bond lengths (Table 3 ▸) observed in the limited number of crystal structures of com­pounds featuring a C=O—AsF_5_ linkage, involving acyl fluorides (Bayer *et al.*, 2021 ▸; Steiner *et al.*, 2024*b* ▸). The As atom resides slightly out of the C_2_C=O plane [0.417 (2) Å]. The C=O—As bond angle [133.83 (8)°] is com­parable to those in other com­pounds where a C=O group acts as a ligand to AsF_5_ (Table 3 ▸). The As—F_ax_ (ax is axial) bond *trans* to As—O is somewhat shorter [1.6890 (8) Å] than the As—F_eq_ (eq is equatorial) bonds in the AsF_5_ moiety [1.6960 (10)–1.7018 (9) Å]. The O—As—F_ax_ angle [176.30 (5)°] is close to linear. The acute O—As—F_eq_ angles [83.84 (5)–89.06 (5)°], the obtuse F_ax_—As—F_eq_ angles [92.94 (5)–94.16 (5)°] and the *trans*-F_eq_—As—F_eq_ angles deviating from linearity [171.85 (5) and 172.89 (5)°] reflect the smaller bond domain (Gillespie, 2008 ▸) and lower bond order (Stuart *et al.*, 2019 ▸) of the As—O bond com­pared to the As—F bonds.

**Figure 6:**
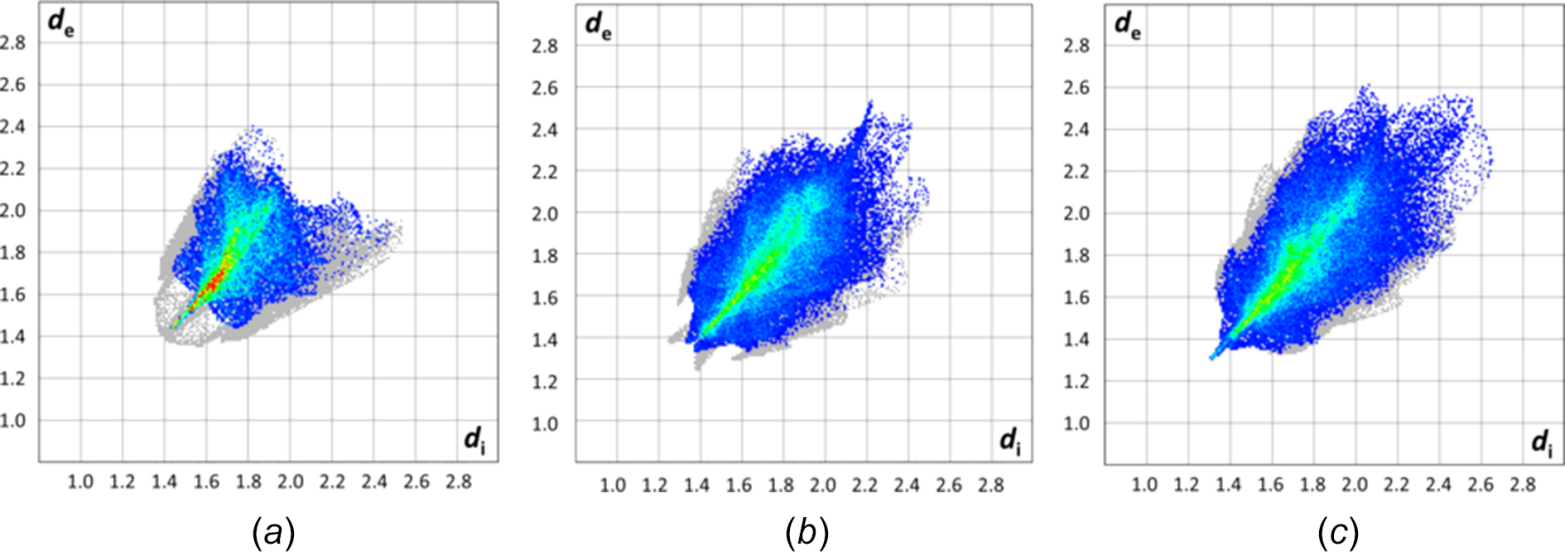
Hirshfeld fingerprint plots of (*a*) (C_6_F_5_)_2_CO (56.8%), (*b*) (C_6_F_5_)_2_COH^+^[AsF_6_]^−^ (69.6%) and (*c*) (C_6_F_5_)_2_CO·AsF_5_ (72.8%), showing the prevalent F⋯F inter­molecular contacts.

In all three crystal structures, *i.e.* (C_6_F_5_)_2_CO, (C_6_F_5_)_2_COH^+^[AsF_6_]^−^ and (C_6_F_5_)_2_CO·AsF_5_, the dominant inter­molecular contacts are F⋯F (Figs. 6 ▸ and S5), which involve contributions of 56.8, 69.6 and 72.8%, respectively, whereas the C⋯F contacts (Fig. S6) cover 30.7, 23.7 and 25.2% of the surface area, respectively. The similarity in the inter­molecular inter­actions between (C_6_F_5_)_2_COH^+^[AsF_6_]^−^ and (C_6_F_5_)_2_CO·AsF_5_ is evi­dent from a com­parison of their Hirshfeld fingerprint plots (Figs. 6 ▸ and S5–S7) (Spackman & McKinnon, 2002 ▸; Spackman *et al.*, 2021 ▸). In the crystal structure of (C_6_F_5_)_2_CO, O⋯F contacts (Fig. S7) also contribute to the packing, accounting for 8.5% of the surface area. However, in the crystal structures of (C_6_F_5_)_2_COH^+^[AsF_6_]^−^ and (C_6_F_5_)_2_CO·AsF_5_, this contribution is reduced to 4.4 and 1.9%, respectively, as the O atom is bonded to hy­dro­gen and arsenic, respectively. In the proton­ated salt, F⋯H contacts involve 2.3% of the surface area (Fig. S8).

## Conclusion

In this work, the syntheses, crystal structure determination and Raman spectra of a protonated deca­fluoro­benzo­phenone salt, (C_6_F_5_)_2_COH^+^[AsF_6_]^−^, and a Lewis acid–base adduct, (C_6_F_5_)_2_CO·AsF_5_, are reported. The crystal structure of (C_6_F_5_)_2_CO was also redetermined at 100 K. These crystal structures represent rare examples of a protonated perfluorinated aro­matic ketone and a ketone–arsenic penta­fluoride adduct.

## Supplementary Material

Crystal structure: contains datablock(s) pfbp_1, pfbphas_1, pfbpasf5_1_1, pfpah_asf6_3_1, global. DOI: 10.1107/S2053229625007697/op3037sup1.cif

Structure factors: contains datablock(s) pfbp_1. DOI: 10.1107/S2053229625007697/op3037pfbp_1sup2.hkl

Structure factors: contains datablock(s) pfbphas_1. DOI: 10.1107/S2053229625007697/op3037pfbphas_1sup3.hkl

Structure factors: contains datablock(s) pfbpasf5_1_1. DOI: 10.1107/S2053229625007697/op3037pfbpasf5_1_1sup4.hkl

Structure factors: contains datablock(s) pfpah_asf6_3_1. DOI: 10.1107/S2053229625007697/op3037pfpah_asf6_3_1sup5.hkl

Additional figures. DOI: 10.1107/S2053229625007697/op3037sup6.pdf

CCDC references: 2483527, 2483526, 2483525, 2483524

## Figures and Tables

**Table 1:** Experimental details Experiments were carried out at 100 K using a Rigaku OD XtaLAB Synergy-S Dualflex diffractometer with an Eiger2 R CdTe 1M detector. The absorption correction was Gaussian (*CrysAlis PRO*; Rigaku OD, 2025 ▸).

	**(C_6_F_5_)_2_CO**	**(C_6_F_5_)_2_COH^+^[AsF_6_]^−^**	**(C_6_F_5_)_2_CO·AsF_5_**
Crystal data			
Chemical formula	C_13_F_10_O	C_13_HF_10_O^+^AsF_6_^−^	C_13_AsF_15_O
*M*_r_	362.13	552.06	532.05
Crystal system, space group	Monoclinic, *C*2	Monoclinic, *P*2_1_/*n*	Orthorhombic, *Pbca*
*a*, *b*, *c* (Å)	20.1460 (4), 5.39987 (10), 5.47058 (14)	8.19207 (7), 21.17409 (18), 9.28475 (9)	12.39109 (18), 12.04038 (17), 20.8754 (3)
α, β, γ (°)	90, 95.912 (2), 90	90, 98.2086 (8), 90	90, 90, 90
*V* (Å^3^)	591.96 (2)	1594.03 (2)	3114.47 (8)
*Z*	2	4	8
Radiation type	Cu *K*α	Cu *K*α	Ag *K*α, λ = 0.56087 Å
μ (mm^−1^)	2.18	4.70	1.25
Crystal size (mm)	0.43 × 0.1 × 0.07	0.09 × 0.05 × 0.03	0.58 × 0.29 × 0.22

Data collection			
*T*_min_, *T*_max_	0.491, 1.000	0.842, 1.000	0.290, 1.000
No. of measured, independent and observed [*I* > 2σ(*I*)] reflections	10783, 1212, 1193	26147, 3336, 3134	97292, 8537, 7132
*R*_int_	0.056	0.032	0.033
(sin θ/λ)_max_ (Å^−1^)	0.629	0.630	0.914

Refinement			
*R*[*F*^2^ > 2σ(*F*^2^)], *wR*(*F*^2^), *S*	0.034, 0.095, 1.11	0.025, 0.066, 1.05	0.031, 0.081, 1.09
No. of reflections	1212	3336	8537
No. of parameters	110	285	271
No. of restraints	1	0	0
H-atom treatment	–	All H-atom parameters refined	–
Δρ_max_, Δρ_min_ (e Å^−3^)	0.28, −0.24	0.30, −0.49	0.53, −0.64
Absolute structure	Flack *x* determined using 514 quotients [(*I*^+^) − (*I*^−^)]/[(*I*^+^) + (*I*^−^)] (Parsons *et al.*, 2013 ▸)	–	–
Absolute structure parameter	0.05 (10)	–	–

Computer programs: *CrysAlis PRO* (Rigaku OD, 2025 ▸), *olex2.solve* (Bourhis *et al.*, 2015 ▸), *SHELXL* (Sheldrick, 2015 ▸), *DIAMOND* (Brandenburg, 2022 ▸) and *OLEX2* (Dolomanov *et al.*, 2009 ▸).

**Table 2:** Hydrogen-bond geometry (Å, °) for (C_6_F_5_)_2_COH^+^[AsF_6_]^−^

*D*—H⋯*A*	*D*—H	H⋯*A*	*D*⋯*A*	*D*—H⋯*A*
O1—H1⋯F16	0.90 (3)	1.63 (3)	2.5279 (16)	178 (3)

**Table 3:** Selected geometric parameters (Å, °) for crystallographically characterized com­pounds featuring AsF_5_ coordinated to a carbonyl group S_2_N_2_CO = 5-oxo-1,3λ^4^,2,4-di­thia­diazole, C_4_H_2_F_2_O_2_ = fumaryl fluoride, CH_2_ClCFO = chloro­acetyl fluoride and CH_2_FCFO = fluoro­acetyl fluoride.

Compound (moiety)	C=O	O—As	As—F_ax_	C—O—As	Reference
(C_6_F_5_)_2_CO·AsF_5_	1.2526 (15)	1.9897 (10)	1.6890 (8)	133.83 (8)	This work
S_2_N_2_CO·AsF_5_	1.279 (4)	1.879 (7)	1.695 (6)	127.3 (1)	Gieren *et al.* (1980 ▸)
C_4_H_2_F_2_O_2_·4(C_4_H_2_F_2_O_2_·AsF_5_)·(C_4_H_2_F_2_O_2_·2AsF_5_)					Bayer *et al.* (2021 ▸)
(C_4_H_2_F_2_O_2_·AsF_5_)	1.221 (2)	2.0367 (15)	1.6840 (14)	132.33 (15)	
(C_4_H_2_F_2_O_2_·AsF_5_)	1.220 (2)	2.0472 (14)	1.6835 (13)	130.67 (14)	
(C_4_H_2_F_2_O_2_·2AsF_5_)	1.215 (3)	2.0444 (14)	1.6926 (13)	130.83 (14)	
CH_2_ClCFO·AsF_5_	1.213 (3)	2.0418 (16)	1.6854 (14)	128.96 (17)	Steiner *et al.* (2024*b* ▸)
CH_2_FCFO·AsF_5_	1.210 (3)	2.0306 (15)	1.6835 (13)	130.49 (15)	Steiner *et al.* (2024*b* ▸)
