# Supplementary material for: Protonated deca­fluoro­benzo­phenone and the deca­fluoro­benzo­phenone–arsenic penta­fluoride adduct
Source: Acta Crystallogr C Struct Chem. 2025 Sep 23;81(Pt 10):577–83. doi: 10.1107/S2053229625007697 (PMC12497096; doi:10.1107/S2053229625007697)
Supplement: Supplementary file 6 [file c-81-00577-sup6.pdf]

STRUCTURAL CHEMISTRY

Volume 81 (2025)

Supporting information for article:

Protonated decafluorobenzophenone and the decafluorobenzophenone–arsenic pentafluoride adduct

Erik Uran and Matic Lozinšek

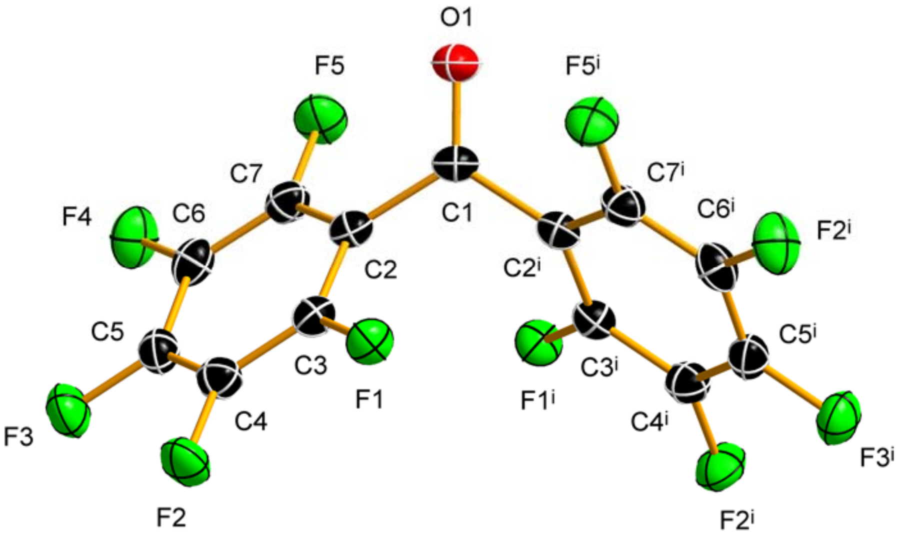

Figure S1 Expanded asymmetric unit with atom-labelling scheme in the crystal structure of $(C_6F_5)_2CO$. Displacement ellipsoids are depicted at the 50% probability level. Symmetry code: (i) $-x + 1, y, -z$.

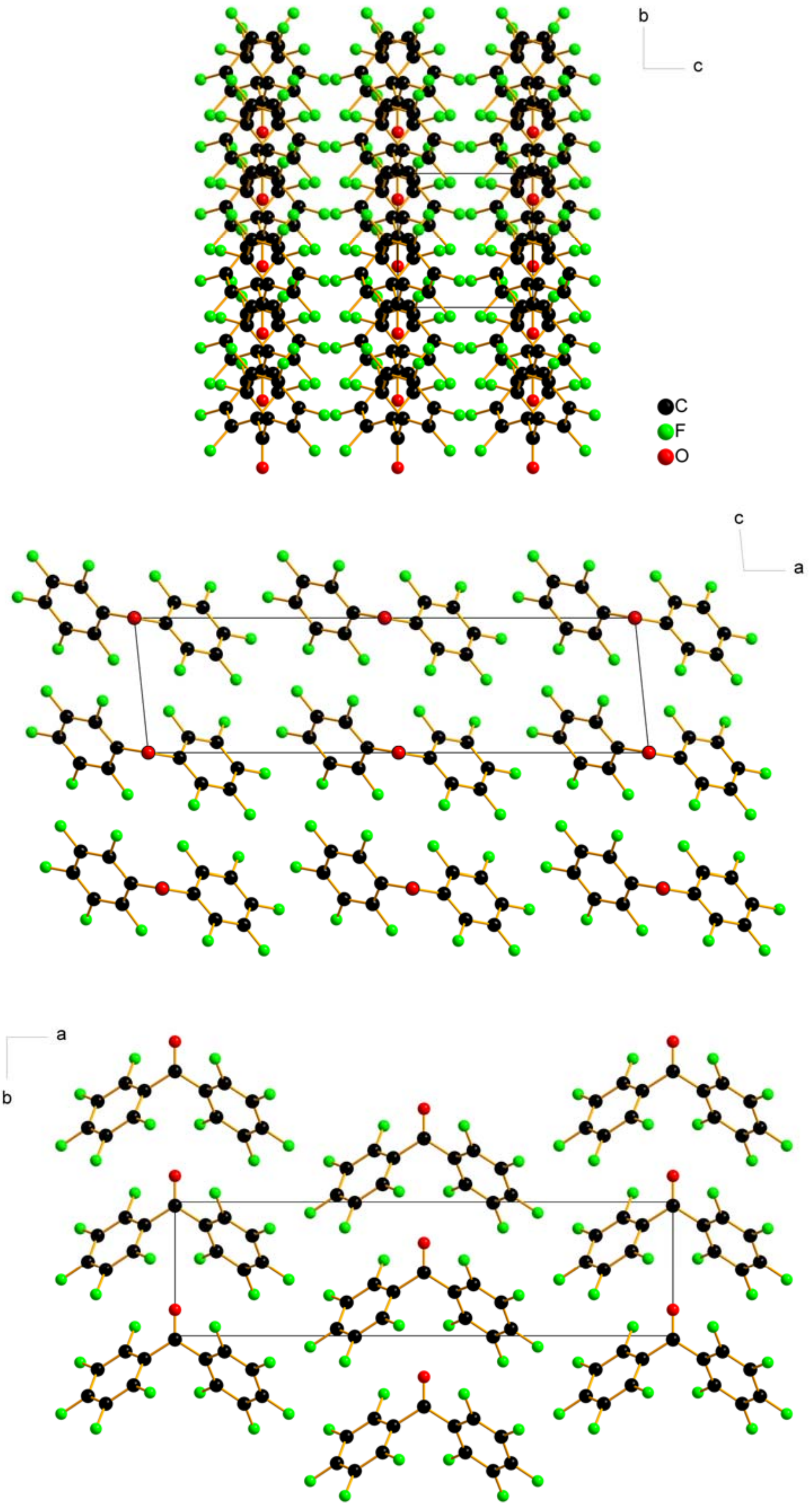

Figure S2 Packing diagrams and the unit cell of the $(C_6F_5)_2CO$ crystal structure viewed along the crystallographic *a*- (top), *b*- (middle) and *c*-axis (bottom).

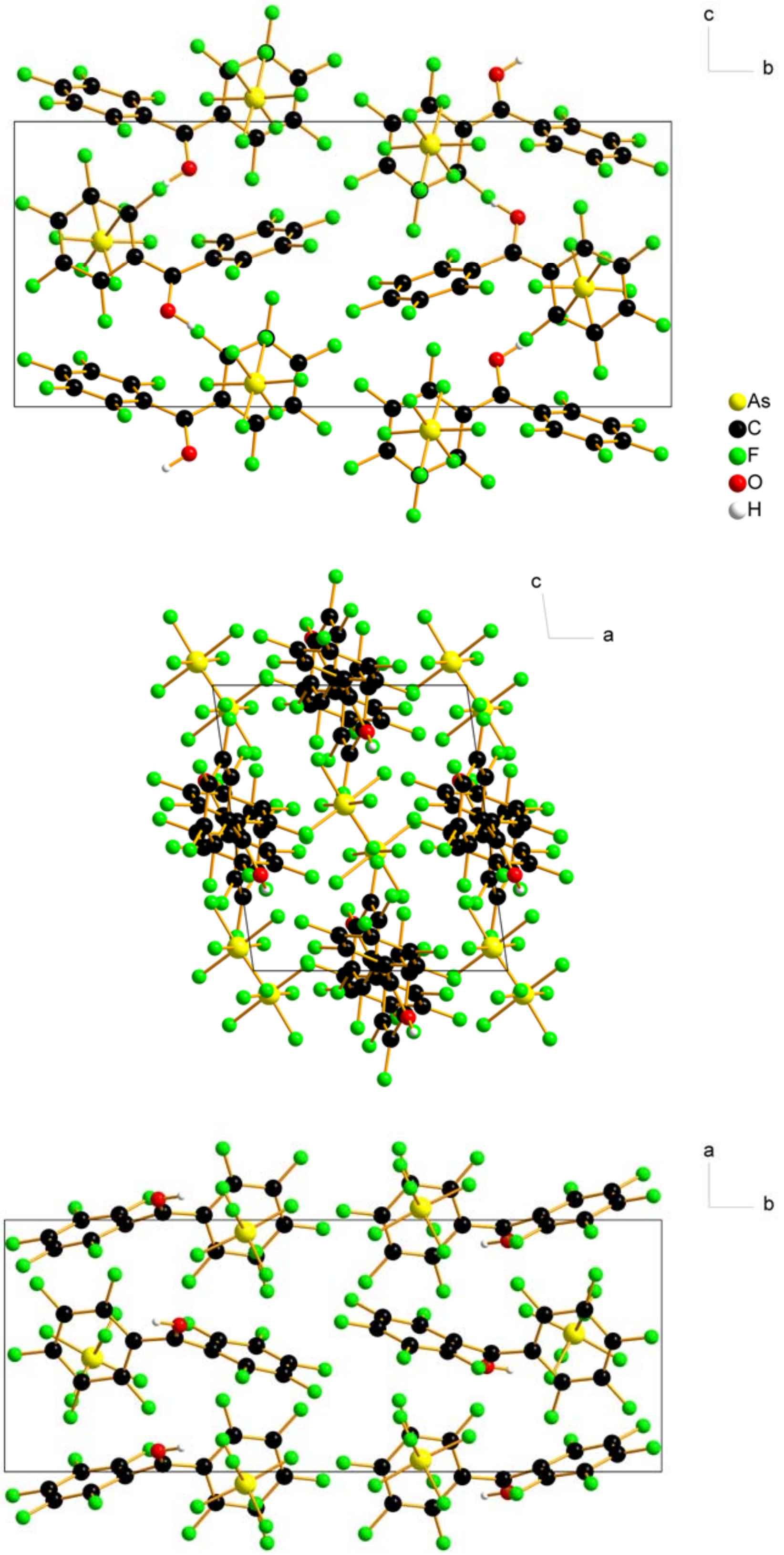

Figure S3 Packing diagrams and the unit cell of the $(C_6F_5)_2COH^+[AsF_6]^-$ crystal structure viewed along the crystallographic *a*- (top), *b*- (middle) and *c*-axis (bottom).

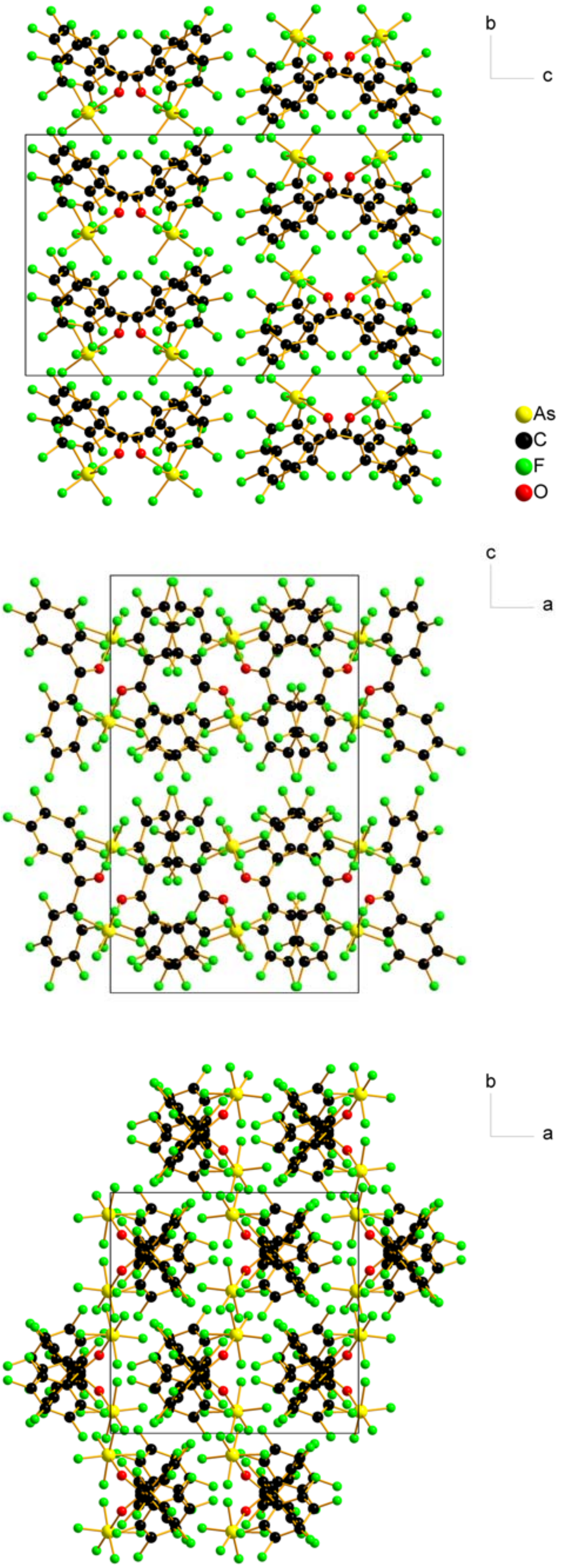

Figure S4 Packing diagrams and the unit cell of the $(C_6F_5)_2CO{\cdot}AsF_5$ crystal structure viewed along the crystallographic *a*- (top), *b*- (middle) and *c*-axis (bottom).

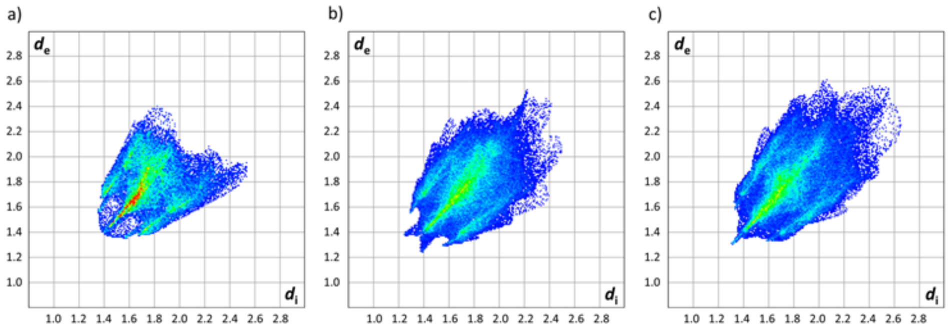

Figure S5 Hirshfeld fingerprint plots of (a) $(C_6F_5)_2CO$, (b) $(C_6F_5)_2COH^+[AsF_6]^-$, and (c) $(C_6F_5)_2CO{\cdot}AsF_5$.

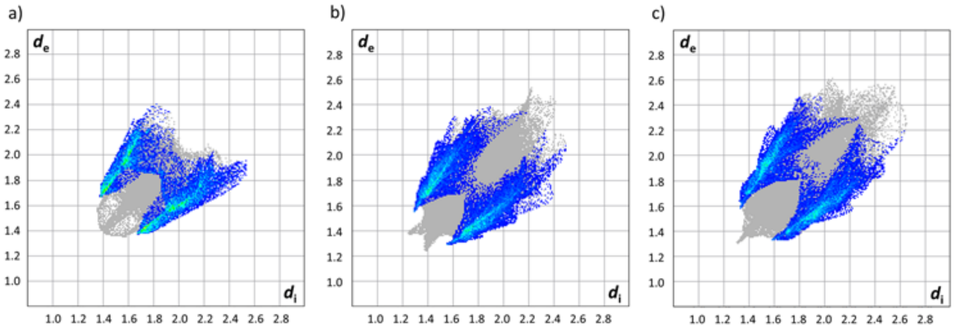

Figure S6 Hirshfeld fingerprint plots for C···F intermolecular contacts of (a) $(C_6F_5)_2CO$ (30.7%), (b) $(C_6F_5)_2COH^+[AsF_6]^-$ (23.7%), and (c) $(C_6F_5)_2CO{\cdot}AsF_5$ (25.2%).

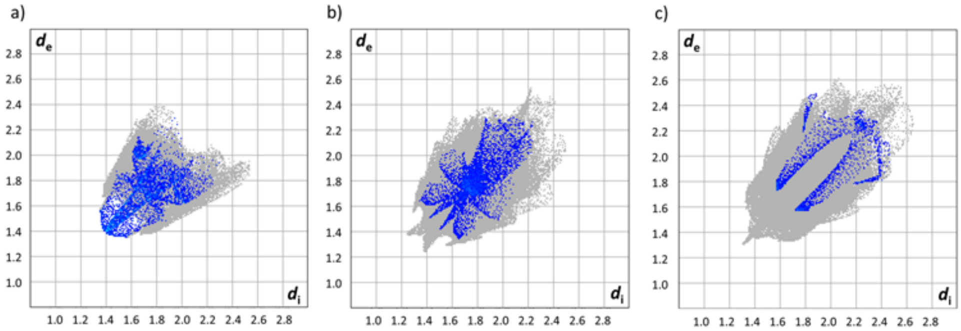

Figure S7 Hirshfeld fingerprint plots for O···F intermolecular contacts of (a) $(C_6F_5)_2CO$ (8.5%), (b) $(C_6F_5)_2COH^+[AsF_6]^-$ (4.4%), and (c) $(C_6F_5)_2CO{\cdot}AsF_5$ (1.9%).

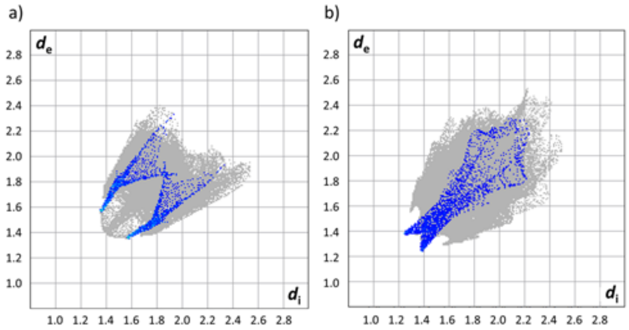

Figure S8 Hirshfeld fingerprint plots for C···O intermolecular contacts of (a) $(C_6F_5)_2CO$ (3.9%) and for H···F contacts of (b) $(C_6F_5)_2COH^+[AsF_6]^-$ (2.3%).
